# Feeling powerful and less emotional: how sense of power reduces aversion to AI services

**DOI:** 10.3389/fpsyg.2026.1760538

**Published:** 2026-04-21

**Authors:** Shengru Ren, Ying Hu

**Affiliations:** Department of Marketing, Renmin University of China, Beijing, China

**Keywords:** AI aversion, artificial intelligence, emotional reliance, evolutionary mismatch, sense of power

## Abstract

**Introduction:**

Despite the growing capabilities of artiffcial intelligence (AI), individuals often resist AI-based services even when these systems outperform human providers. This research examines how individuals’ sense of power inffuences AI aversion. From an evolutionary perspective, we conceptualize AI aversion as an emotional response that reffects a mismatch between social–cognitive mechanisms evolved for human interaction and the emergence of artiffcial decision-makers. We argue that feeling powerful shifts individuals away from affective, evolution-arily grounded vigilance toward more deliberative, outcome-focused evaluations of AI services, thereby attenuating resistance rooted in species-based categorization.

**Methods:**

We conducted experiments (*N* = 1240) to examine the effect of sense of power on AI aversion. We employed multiple methods to manipulate participants’ sense of power, specifically including an open-ended writing task and a semantic manipulation approach. In addition, we measured attitudes toward AI across three distinct service contexts: medical, financial, and dating services.

**Results:**

Study 1 shows that inducing a sense of power decreases individuals’ reluctance to adopt AI services. Study 2 replicates this effect using a subtle yet practically relevant power manipulation. Study 3 provides process evidence, demonstrating that feeling powerful reduces reliance on feelings in the decision-making process, thereby lowering AI aversion. Together, these ffndings suggest that heightened power fosters less emotional decision-making, which in turn mitigates resistance to AI.

**Discussion:**

This research contributes to the literature on AI adop-tion and sense of power by identifying a psychologically grounded interven-tion that reduces AI aversion without altering AI’s functional attributes, offering actionable insights for service providers and policymakers seeking to increase individual openness to AI services.

## 1 Introduction

Artificial intelligence (AI) is becoming increasingly integrated into various consumer services, such as hospitality (Chan, 2026), restaurants (Lu et al., 2024), public services (Longoni et al., 2023), product recommendation (Longoni and Cian, 2022), customer service (Garvey et al., 2023; Jin et al., 2025; Wang et al., 2025), and healthcare (Dai and Singh, 2025; Gaczek et al., 2023; Huo et al., 2023). AI can be embodied or invisible, spanning interactive agents (e.g., service robots, avatars, chatbots, and voice assistants) and algorithmic systems (e.g., recommendation engines and automated triage decision tools) that operate within platforms and service processes. The global AI market is projected to reach $990 billion by 2027 (Bain & Company, 2024), underscoring AI’s growing relevance to individuals. In practice, AI systems are already widely adopted in service and marketing contexts. For instance, healthcare organizations deploy tools, such as Aidoc^1^ and Babylon Health^2^; financial institutions rely on AI-advisors, such as Betterment^3^ and Wealthfront^4^; and dating apps, such as Tinder^5^ and Hinge^6^, use matching algorithms to recommend potential partners. These developments make understanding individuals’ responses to AI service providers particularly important. However, despite its technological sophistication and expanding presence, individual aversion to AI remains a significant barrier to widespread adoption, suggesting that resistance to AI may not be fully explained by functional limitations or performance concerns alone.

AI aversion can have far-reaching consequences. It undermines individual trust and adoption intention, limits the efficiency and scalability of AI-driven services, and creates economic inefficiencies for firms that invest heavily in AI development but face slow market acceptance, effects that are particularly pronounced in high-stakes contexts (Filiz et al., 2023). Supporting this, Longoni et al. (2023) show that when an AI system fails, such failures are generalized more broadly than comparable human errors due to “algorithmic transference,” whereby people perceive AI systems as a homogeneous category and consequently transfer failure information from one AI to others. As a result, a single AI malfunction can erode trust not only in the specific system but also in the broader institutions that deploy it. On a societal level, persistent AI aversion may impede technological innovation diffusion, increase human workload, and exacerbate service inequality, as individuals reluctant to engage with AI miss out on convenience and personalized benefits. Thus, understanding and mitigating AI aversion is both a theoretical and practical imperative.

Scholars have extensively explored why individuals resist AI, revealing that this resistance often stems from emotional discomfort rather than rational distrust (Kyung and Kwon, 2022). For example, although AI-generated messages are often perceived as more empathetic than human-generated ones, recipients report feeling less heard upon learning the message came from AI (Yin et al., 2024). Similarly, despite evidence that AI doctors perform better than humans, patients continue to prefer human doctors because of perceived uniqueness neglect (Longoni et al., 2019). Emerging research suggests that AI aversion may also reflect a form of speciesism, whereby AI agents are categorically devalued simply because they do not belong to the human species (Schmitt, 2020). From an evolutionary perspective, such species-based bias can be understood as a byproduct of social–cognitive mechanisms evolved to distinguish ingroup from outgroup agents, which prioritize rapid, affective responses when encountering non-human entities (Cosmides and Tooby, 1992; Li et al., 2018). Together, these findings underscore that individuals’ emotional attachment to human interaction often overrides rational assessments of performance and reliability, raising the possibility that such emotional reactions may be rooted in deeper, evolutionarily shaped social–cognitive processes rather than purely reflective evaluations of technology.

As a result, even highly capable or human-like AI may evoke emotional resistance once their artificial identity becomes salient (Yin et al., 2024). In efforts to reduce AI aversion, most existing literature has focused on enhancing AI’s objective performance rather than addressing the human decision-making process itself. Prior studies have attempted to mitigate AI aversion by improving AI’s functionality, such as increasing transparency (Cadario et al., 2021; Mourali et al., 2025), enhancing customization options (Chen et al., 2024; Longoni et al., 2019), or emphasizing AI’s ability to learn and adapt (Berger et al., 2021). However, it remains unclear when and how such evolutionarily grounded, affective resistance can be mitigated, particularly without altering AI’s functional features.

Therefore, one important question arises: can affective-driven resistance toward AI be attenuated by situational psychological conditions during service evaluation, even when AI’s functional attributes remain unchanged? To answer this question, we draw upon the concepts of sense of power and lay rationalism to explore a new psychological pathway for reducing individual resistance toward AI service. Building on evolutionary accounts of AI aversion, we propose that resistance to AI stems from intuitive emotional responses that evolved for human–human interaction but do not translate well to interactions with artificial agents. Importantly, such responses are not inherently rigid. The notion of lay rationalism captures the relative weight individuals place on reason versus feelings in decisions that involve trade-offs between the two factors (Hsee et al., 2015). Accordingly, affective resistance to AI may be attenuated by situational cues that shift individuals away from affective processing toward more analytical, outcome-focused evaluation. Building on the agentic-communal framework of power (Rucker et al., 2012), individuals with a high sense of power are more agentic and outcome-focused, prioritizing goal attainment and instrumental reasoning over affective considerations. In contrast, those with a low sense of power are more communal-oriented and more attentive to emotional cues. Thus, we propose that experiencing a higher sense of power tends to rely less on feeling in decision-making, thereby exhibiting lower aversion toward AI services rooted in emotional discomfort and species-based categorization.

This research makes several important contributions to the literature on AI attitude (Dang and Li, 2025; Qin et al., 2025; Yin et al., 2024) and power psychology (Chen et al., 2001; Fiske, 1993; Kipnis, 1972). By integrating evolutionary perspectives on social cognition with power psychology, this research reframes AI aversion as a malleable affective response rather than a fixed opposition to technology. First, we identify a psychological intervention that can effectively reduce AI aversion. Rather than focusing on external technological features or message framing (Chen et al., 2024; Hou et al., 2024; Huo et al., 2023; Mourali et al., 2025), this research highlights how psychological state in decision-making can be shaped to reduce AI aversion. Second, we enrich the sense of power literature by proposing a main effect of sense of power on judgment and decision-making, extending beyond its traditional role as a contextual moderator in AI adoption (Wang et al., 2025; Zhang and Song, 2024). While previous research has predominantly emphasized the negative consequences of high power (e.g., egocentrism and reduced empathy; Galinsky et al., 2003, 2006), our findings highlight a positive outcome: reduced emotional bias in AI decision contexts.

From the practical viewpoint, the findings suggest that AI service designers and marketers can mitigate individual resistance by activating or simulating a sense of power during the decision process. Encouraging users to adopt a rational evaluation mindset and rely less on feelings during decision-making can effectively reduce affect-driven aversion toward AI systems. These insights offer actionable strategies for promoting AI service adoption and improving human–AI collaboration in marketing and service environments.

## 2 Theoretical background and hypotheses

### 2.1 Affective foundations of AI aversion

Artificial intelligence (AI) refers to the ability of machines or systems to perform tasks traditionally requiring human intelligence, such as learning, reasoning, and decision-making (De Freitas et al., 2023). AI spans from intangible algorithms, such as generative models like ChatGPT, to tangible robots, such as versatile humanoid robots like Tesla’s Optimus (Qin et al., 2025). AI technologies are becoming increasingly embedded in individuals’ daily encounters across diverse domains. In hospitality settings, service robots routinely perform tasks such as delivering meals to guest rooms or handling customer requests (Chan, 2026). In restaurants, AI-based systems support menu recommendations and assist with food preparation (Lu et al., 2024). In healthcare environments, AI-enabled diagnostic tools and surgical robots are employed to aid clinicians in performing complex procedures and monitoring patient conditions (Dai and Singh, 2025; Gaczek et al., 2023). In elder-care contexts, socially assistive robots are used to provide companionship, support daily activities, and supplement caregiver support (Olatunji et al., 2024; Veling and Villing, 2024). Similarly, across customer service sectors, AI-driven chatbots and virtual agents increasingly manage routine inquiries and service requests (Jin et al., 2025; Wang et al., 2025).

Despite the rapid diffusion of AI across consumer services, individuals frequently exhibit AI aversion, defined as a tendency to exhibit more negative attitudes and behaviors toward AI relative to humans, even when AI demonstrates comparable or superior performance (Cadario et al., 2021; Longoni et al., 2019). For instance, in medical care, the general public strongly prefers human medical professionals to make medical decisions both now and “100 years from now.” (Rojahn et al., 2023). Similarly, in public service settings, cities using algorithms may be perceived as less resident-friendly because they evoke a sense of alienation and reduce perceived city friendliness (Oleksy et al., 2023; Tomasz et al., 2023).

Existing research suggests that AI aversion is driven by both cognitive and emotional factors. From a cognitive perspective, individuals express concerns about AI’s opacity (Cadario et al., 2021), rigidity (Berger et al., 2021), and restricted learning capabilities (Longoni and Cian, 2022). However, affective responses—such as emotional discomfort (Kyung and Kwon, 2022), distrust toward non-human agents (Schmitt, 2020), perceived lack of empathy (Longoni and Cian, 2022), and the belief that AI cannot recognize individual uniqueness (Longoni et al., 2019)—play a more dominant role in fostering AI aversion. Crucially, these affective reactions persist even when AI’s objective performance advantages are made salient. For example, while interventions addressing cognitive concerns, such as enhancing explainability or emphasizing AI’s learning potential, can reduce some forms of distrust, they fail to fully mitigate resistance (Dietvorst et al., 2018). Similarly, individuals may acknowledge that AI doctors outperform human physicians yet still prefer human providers due to feelings of unease or concerns about being insufficiently understood as unique individuals (Longoni et al., 2019). Recipients report feeling less heard once the artificial origin of the message is revealed, although AI-generated messages can be perceived as empathetic in content (Yin et al., 2024). Taken together, these findings suggest that AI aversion is not merely a result of rational assessments; rather, it is deeply rooted in individuals’ emotionally grounded response, which are activated early and often dominates subsequent evaluation of AI systems.

Evolutionary mismatch theory posits that psychological tendencies shaped by ancestral environments may continue to guide judgment in modern contexts for which they were not originally designed (Li et al., 2018). Human social cognition evolved in environments in which socially relevant agents were human, whereas non-conspecific entities often signaled uncertainty or potential threat. As a result, humans developed cognitive systems specialized for distinguishing “us” from “them,” which prioritize rapid, affective responses over deliberative reasoning when encountering unfamiliar or categorically distinct agents (Cosmides and Tooby, 1992). Related research on the behavioral immune system further demonstrates that entities perceived as non-self can automatically elicit intuitive emotional reactions, such as discomfort, distrust, and avoidance, functioning as heuristics for self-protection (Schaller and Park, 2011). These affective responses are largely automatic and operate prior to conscious reflection, shaping downstream judgments and preferences.

One influential account conceptualizes AI aversion as a manifestation of speciesism, defined as a categorical bias whereby artificial agents are assigned lower moral standing, legitimacy, or trust simply because they do not belong to the human species (Schmitt, 2020). From this perspective, resistance to AI reflects a form of species-based discrimination rather than a reasoned assessment of technological capability. Consistent with this account, studies show that even emotionally capable or highly human-like AI may be evaluated favorably at first, yet become aversive once its artificial identity is revealed (Yin et al., 2024). Empirical evidence further shows that individuals high in speciesism are less willing to accept AI in independent roles that require autonomous authority, such as medical decision-making, but are more accepting of AI in assistive roles that preserve human oversight and control (Huo et al., 2023).

Thus, species-based bias toward AI can be understood as a byproduct of evolved social–cognitive mechanisms specialized for managing interactions with biological conspecifics. Although AI systems can display high levels of competence, responsiveness, and even emotional cues, their non-human identity places them outside the evolved boundaries of moral and social consideration, leading to intuitive, immediate emotional resistance rather than reflective evaluation (Haslam, 2006; Gray et al., 2007). Together, these perspectives suggest that AI aversion reflects species-based biases grounded in evolutionarily shaped social categorization, rather than a uniform rejection of artificial agents. This raises a critical question for understanding AI–human interaction: under what psychological conditions do such biases exert greater or lesser influence on evaluative judgment? In the following section, we propose that a sense of power is one such condition that systematically alters how individuals approach decisions involving AI services.

### 2.2 Sense of power and AI adoption

Sense of power is generally defined as an individual’s subjective perceived ability to influence others or control valuable resources (Keltner et al., 2003; Anderson et al., 2012). Extensive research shows that power systematically shapes cognition, motivation, and interpersonal behavior (Anderson et al., 2012; Dubois et al., 2016) and serves as a context-sensitive psychological state that influences a wide range of individual choices (Liang et al., 2023). Sense of power can be understood as a situational cue that shapes how individuals approach evaluative judgments, such as the extent to which affective versus instrumental considerations guide decision-making. Within the agentic-communal model of power (Rucker et al., 2012), high-power individuals tend to adopt an agentic orientation, focusing on goals, instrumental outcomes, and competence-related information. Low-power individuals, in contrast, adopt a communal orientation, attending to relational cues, warmth, and social harmony. This distinction is reflected in consumption preferences: high-power individuals evaluate service providers through performance and competence (Liang et al., 2023; Zhang et al., 2021), whereas low-power individuals rely more on warmth signals such as politeness and friendliness (Dubois et al., 2016; Zhang and Song, 2024).

Power also shapes emotional processing and regulation. High-power individuals are more likely to engage in cognitive reappraisal, a strategy that involves reframing a situation objectively to reduce emotional impact (Gross and John, 2003; Leach and Weick, 2020). This tendency to downweight emotional input aligns with findings that high-power individuals view others more instrumentally—as means to achieve goals rather than as relational partners (Gruenfeld et al., 2008). In contrast, low-power individuals are more emotionally sensitive and more attuned to interpersonal cues. Importantly, we do not argue that high-power individuals experience less affective discomfort toward AI. Rather, prior research suggests that individuals with greater power rely less on affective cues when forming judgments. Thus, even if AI evokes affective discomfort broadly, high-power individuals may place less weight on these affective responses when evaluating AI services. Taken together, these findings suggest that individuals with a higher sense of power rely less on emotional information during evaluative judgment, thereby reducing the influence of emotionally grounded biases, such as those underlying AI aversion.

**Figure 1:**
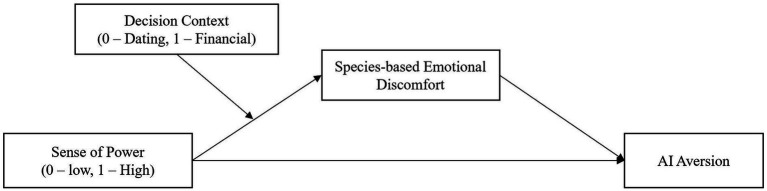
Conceptual framework.

If high-power individuals rely less on emotional input, how does this shape their evaluation of AI services? As AI aversion is fundamentally rooted in species-based affective discomfort toward non-human agents, individuals with a higher sense of power should exhibit lower aversion to AI services. When applied to AI adoption, this reasoning suggests a novel insight: because AI is more likely to be construed instrumentally rather than relationally, high-power individuals should exhibit less aversion to AI services. Their exchange orientation reduces reliance on emotional considerations and increases focus on service effectiveness, regardless of whether the provider is human or non-human. Conversely, low-power individuals’ communal orientation heightens their reliance on relational cues, leading them to resist AI, which lacks warmth and emotional responsiveness. Taken together, these arguments lead to the following hypotheses (see Figure 1 for the conceptual framework):

*H_1_*: Experiencing a higher (vs. lower) sense of power reduces individuals’ aversion to AI services.

*H_2_*: The effect of situational power on AI aversion is mediated by individuals’ relative reliance on emotion versus rationality in decision-making. Specifically, experiencing higher (vs. lower) power decreases reliance on emotional thinking, which in turn lowers AI aversion.

Importantly, decision contexts differ in the extent to which affective responses toward social agents are likely to be activated. Within service settings, tasks perceived as objective are typically aligned with cognitive processing, whereas subjective tasks are more closely related to emotional or affective competencies (Inbar et al., 2010). For example, selecting a financial product would be considered an objective task, while choosing a romantic partner would be categorized as subjective. To further establish the proposed mechanism, we argue that individuals’ reliance on reason versus feeling is contextually malleable: in rational, low-emotion contexts, such as financial decisions, individuals are already prompted to engage in analytical reasoning. In such situations, affective input is minimal for both high- and low-power individuals, and therefore differences in emotional reliance—and consequently AI aversion—should be attenuated. In contrast, emotionally rich contexts, such as dating decisions, heighten the salience of feelings and interpersonal concerns. Under these conditions, differences in emotional reliance between high- and low-power individuals should be most pronounced. High-power individuals, who downregulate emotional input, should show greater acceptance of AI, whereas low-power individuals, whose evaluations are more affectively driven, should display stronger AI aversion. Therefore, decision context should moderate the indirect effect of power on AI aversion through emotional reliance.

*H_3_*: The indirect effect will be stronger in emotionally salient decision contexts and attenuated in rational decision contexts.

## 3 Method

We conducted three studies to test our conceptual model (Table 1 summarizes the studies). Studies 1 and 2 provide causal evidence that a higher sense of power reduces AI aversion in a medical service context. Consistent with prior research (Dietvorst et al., 2015; Longoni et al., 2019), we conceptualize AI aversion as individuals’ reluctance to prefer or accept AI relative to a human decision-maker. To establish the robustness of our findings, we operationalized this construct using multiple measures across studies, such as relative preference between human and AI providers, separate evaluations of AI versus human providers in between-subject designs, and direct assessments of AI acceptance. Specifically, study 1 manipulated the sense of power using a social role-playing task and examined participants’ relative preference for medical service delivered by AI versus human providers. Study 2 adopted a cognitive priming method to activate the feeling of power, and we tested resistance to medical AI by examining the willingness to utilize medical AI and the satisfaction after the medical service compared with human providers. Next, study 3 tested the proposed process using a moderation-of-process approach by varying decision context to infer shifts in reliance on emotional versus rational considerations, examining whether the effect of power on AI acceptance is attenuated in less emotional contexts. Across studies, we applied a common exclusion principle: participants were excluded only if they failed to meaningfully engage with the focal task or failed a pre-specified attention check. In studies using open-ended writing tasks, such as the core manipulation (Studies 1 and 3), this criterion included responses that were clearly irrelevant or incomplete. In Study 2, which used a semantic manipulation rather than an open-ended writing task, no comparable writing-based exclusion criterion was applied.

**Table 1:** Study overview.

Study	Design/manipulation	Context	DV	Key result
Study 1	Between-subjects: power priming [high power (boss) vs. low power (employee)]	Medical	Relative preference for human (vs. AI) doctor	High power reduced AI aversion by lowering preference for the human doctor relative to the AI doctor.
Study 2	2 × 2 between-subjects: subtle power cue [honorific address vs. neutral address] × doctor type [AI vs. human]	Medical	Evaluation of the assigned doctor	High power improved attitudes toward the AI doctor but did not affect attitudes toward the human doctor.
Study 3	2 × 2 between-subjects: power priming [high power (boss) vs. low power (employee)] × service type [dating (emotional) vs. financial (rational)]	Financial vs. Dating	AI acceptance	Power increased AI acceptance only in the emotional (dating) context, consistent with the proposed mechanism.

### 3.1 Study 1: the impact of sense of power on AI aversion

Study 1 provides preliminary evidence for our propositions. It tests whether participants primed to feel powerful show less AI aversion than those primed to feel powerless. Following previous research (Rucker et al., 2012), because of the myriad and various social roles that individuals play within the societal framework, the feeling of power is not just determined by the usual structural differences but can also be changed based on temporary changes. Feeling powerless or powerful can be quickly and simply elicited by assigning an individual to an actual hierarchical role of a boss/employee for a single task (Galinsky et al., 2003).

#### 3.1.1 Procedures

We recruited 240 participants from a Chinese online data collection platform (Credamo^7^), and we excluded 11 participants who did not complete the writing task as instructed (i.e., whose writing content was totally irrelevant to our question). Thus, the final sample size for study 1 was 229 (*M*_age_ = 31.07 years, SD = 9.82; 62% women). We used a between-subjects design and randomly assigned participants to one of two conditions that differed in the sense of power.

To manipulate the sense of power, we asked participants to complete a role-playing writing task. In the high-power condition, participants were asked to imagine themselves as the owner of a company and try to vividly imagine what they would be like as a boss (i.e., how they would feel, think, and act). By contrast, in the low-power condition, participants were asked to imagine themselves as the employee of a company and then finish the writing task. After finishing the writing task, participants were required to report their feelings during the imagination task as a manipulation check. We used three items on a 9-point Likert-type scale anchored at *1 = very powerless/obedient/anxious and 9 = very powerful/dominant/confident* (Cronbach’s *α* = 0.95). As a dependent variable, participants indicated their relative preference for the doctor. Inspired by prior study (Longoni et al., 2019; Zhao and Xiao, 2024), participants were given a medical service situation and asked to report their choice of service provider. To ensure that knowledge about the medical domain was consistent across conditions, participants first read a description of Medical AI. They were then offered a situation in which they decided to conduct a skin cancer screening in the hospital. We provided a short introduction to skin cancer screening to eliminate the impact of unfamiliarity, and then they were asked to report their relative preference for a service provider on an 8-point bipolar scale (*1 = AI doctor and 8 = human doctor*), with higher scores indicating greater AI aversion. Demographic information was collected by the end.

#### 3.1.2 Results

##### 3.1.2.1 Manipulation check

A one-way ANOVA on participants’ feelings of power was significant (*F* (1, 227) = 589.74, *p* < 0.001). Specifically, people who imagined themselves as a boss felt more powerful (*M* = 7.13, SD = 1.03) than people who imagined themselves as an employee (*M* = 2.92, SD = 1.56). Thus, our manipulation of feelings of power was successful.

##### 3.1.2.2 Relative preference for a human (vs. AI) doctor

**Figure 2:**
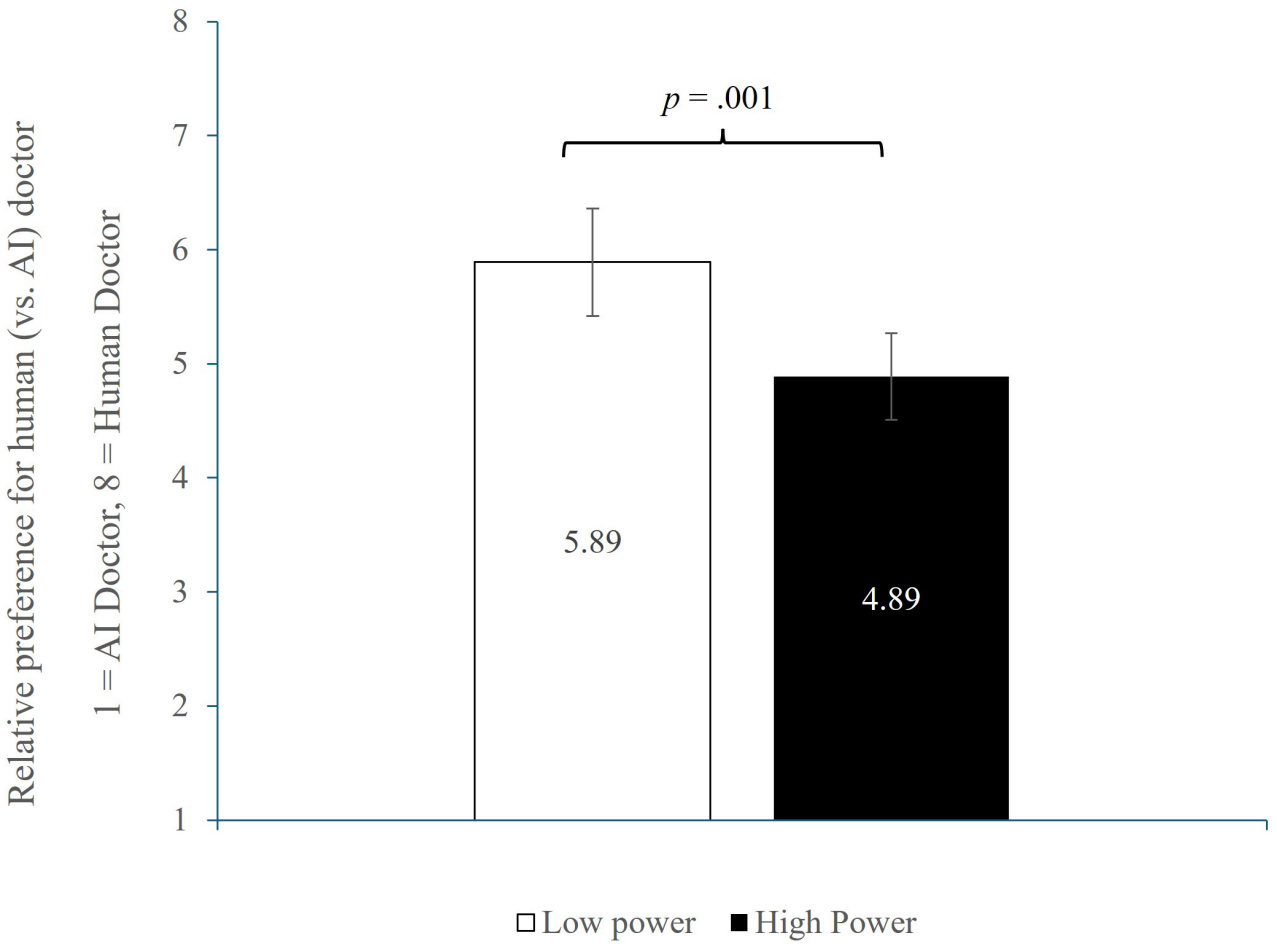
Study 1 result.

Next, we conducted a one-way ANOVA of feelings of power on participants’ relative preference for the medical service providers (see Figure 2). The results revealed a significant effect of power (*F* (1, 227) = 10.54, *p* = 0.001, ηp^2^ = 0.044). As predicted, participants in the high-power condition reported lower preference for the human doctor (*M* = 4.89, SD = 2.55) than those in the low-power condition (*M* = 5.89, SD = 2.10). Given that lower scores reflect greater relative preference for the AI doctor, this result indicates that power attenuated AI aversion.

#### 3.1.3 Discussion

Study 1 provided initial support for our central hypothesis: individuals who feel powerful exhibit less aversion to AI. Although role-playing is an effective method for inducing feelings of power or powerlessness, the boss-versus-employee manipulation may differ on multiple dimensions beyond power, potentially confounding the results. Additionally, it remains unclear whether the effect is driven by high-power or low-power conditions. To address these concerns, study 2 adopts a semantic priming approach that isolates and manipulates the sense of power more cleanly, focusing solely on our key psychological construct of interest, and we compare the high-power condition with a control condition.

### 3.2 Study 2: subtle power manipulation and AI aversion

Study 2 aimed to extend the findings of Study 1 in two important ways. First, we sought to replicate the effect of power on AI aversion using a more subtle and ecologically valid semantic priming method. Sense of power, as a psychological state, can be activated not only through structural roles but also through subtle linguistic cues embedded in social interactions (Galinsky et al., 2003). Building on this insight, we manipulated pronoun use and service-oriented language to induce a sense of power. We examined whether individuals exposed to high-power language would show lower aversion to AI compared to those exposed to neutral language. Second, rather than comparing high-power and low-power conditions as in study 1, we compared a high-power condition with a neutral control condition. This design allowed us to test whether it is the experience of high power that reduces AI aversion, rather than low power inflating it.

#### 3.2.1 Procedures

Study 2 adopted a 2 (provider: AI vs. Human) × 2 (power: high vs. control) between-subjects design. We recruited 600 participants (*M*_age_ = 31.46 years, SD = 8.21; 36.8% men, 63.2% women) on Credamo for monetary compensation, and they were randomly assigned to one of the four conditions.

Under the guise of a role-playing task, all participants were asked to imagine that they were planning to conduct a skin cancer screening in a hospital. Participants were told that their doctor would be either an AI doctor or a human doctor (Dr. Zhang), depending on the doctor’s condition. To manipulate power, we varied the formality and tone of the language used in the scenario. In the high-power condition, participants read materials featuring formal, honorific expressions commonly used in Chinese service settings—specifically, the respectful pronoun “您” (a formal version of “you”) and phrases such as “provide service for you.” In contrast, participants in the control condition received a more neutral version of the same materials using the informal pronoun “你” and phrasing such as “conduct skin cancer screening.” In Mandarin Chinese, the use of “您” versus “你” conveys varying levels of respect and social distance. “您” is typically used to address individuals of higher status or to signal deference, which subtly casts the recipient in a more powerful role. This effect is not unique to the Chinese. Cross-linguistic evidence suggests that many languages—such as Japanese, Korean, French, and German—encode social hierarchy and formality into everyday address forms (e.g., T/V distinctions, honorifics), which shape interpersonal perception and self-construal in similar ways (Brown and Gilman, 1960).

Our dependent variable is the attitude toward the service provider. Our measurements include two steps: first, participants were shown a medical situation where they had recently noticed a black mole on their skin, and planned to conduct the skin cancer screening in a hospital. They were randomly told AI doctor or Dr. Zhang would be their service provider. At this time, they were asked about their acceptance of the AI doctor or Dr. Zhang on a 9-point Likert scale, in which *1 = Totally unaccepted and 9 = Totally accepted*. Secondly, we described the process of skin cancer screening in detail for participants, and except for the wording of power manipulation, all descriptions were the same across conditions. Then they were asked to report their satisfaction with the assigned service provider on a 9-point Likert scale, in which *1 = Totally unsatisfied and 9 = Totally Satisfied.* Health anxiety and AI familiarity (e.g., “How familiar are you with artificial intelligence (AI) technology?,” and “How familiar are you with AI-based medical providers?,” *1 = Not at all and 9 = Extremely*, Cronbach’s *α* = 0.80) were collected at the end for control. We also assessed participants’ subjective sense of power using a three-item scale similar to study 1 (Cronbach’s *α* = 0.77).

#### 3.2.2 Results

##### 3.2.2.1 Manipulation check

A one-way analysis of variance (ANOVA) of the power manipulation on participants’ subjective feeling of power was partially significant (*F* (1, 598) = 3.80, *p* = 0.052, ηp^2^ = 0.006). Specifically, people who were asked honorific words feel more powerful (*M* = 3.68, SD = 1.39) than people who were in the control condition (*M* = 3.46, SD = 1.37).

##### 3.2.2.2 Evaluation of the assigned doctor

**Figure 3:**
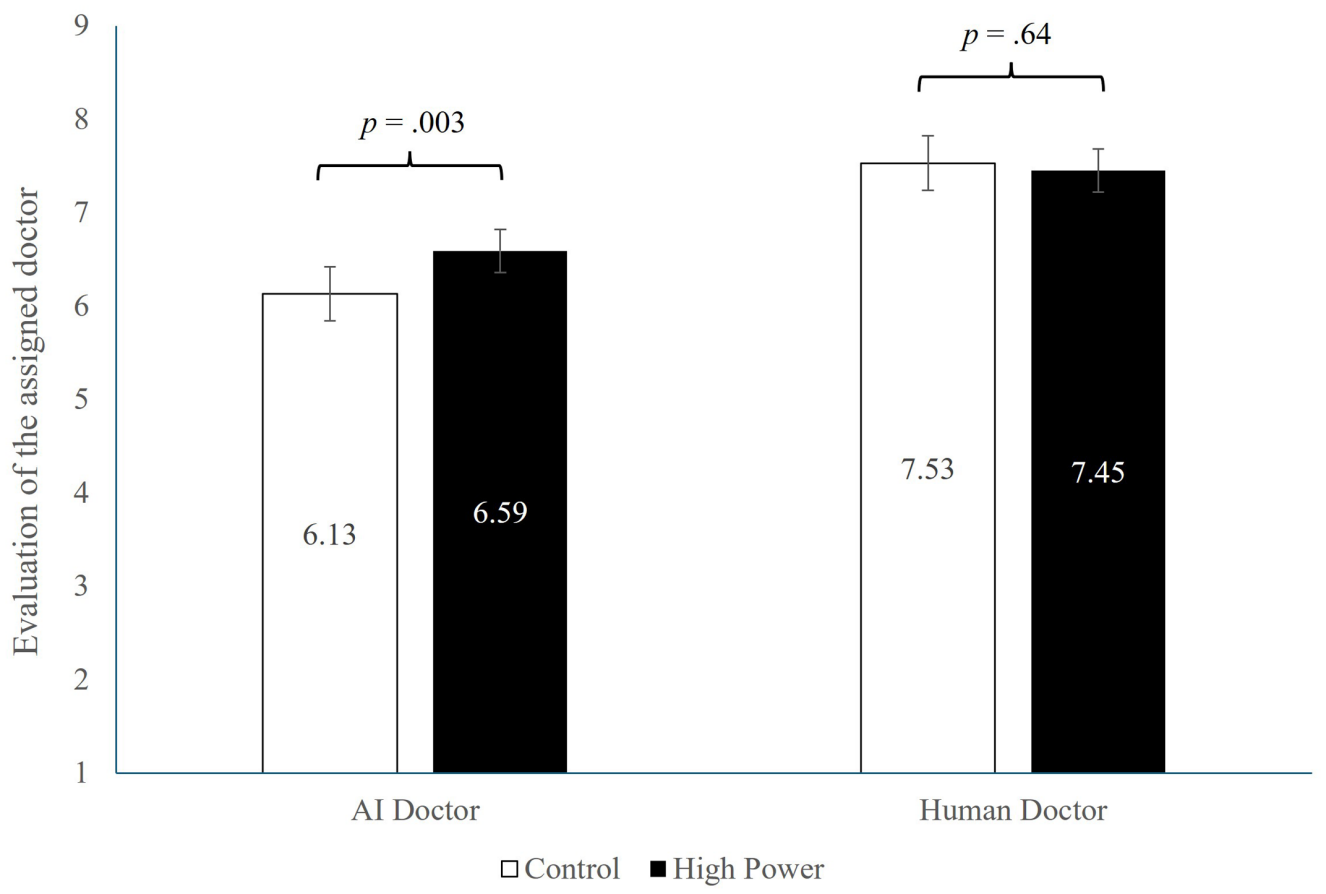
Study 2 result.

A 2 × 2 ANOVA on participants’ evaluations toward the medical service provider (higher values indicate more favorable attitudes) produced a significant main effect of provider type (*F* (1, 596) = 104.17, *p* < 0.001, ηp^2^ = 0.149), with participants generally preferring the human doctor (*M* = 7.49, SD = 1.01) over the AI doctor (*M* = 6.36, SD = 1.64). The main effect of power was not significant (*F* (1, 596) = 3.05, *p* = 0.081, ηp^2^ = 0.005). Importantly, the analysis showed a significant interaction effect (*F* (1, 596) = 5.80, *p* = 0.016, ηp^2^ = 0.010). Planned contrasts revealed that participants who felt more powerful reported more favorable attitudes toward the AI doctor (*M* = 6.59, SD = 1.45) than those in the control condition (*M* = 6.13, SD = 1.79, *F* (1, 596) = 8.63, *p* = 0.003, ηp^2^ = 0.014). In contrast, participants’ attitudes to the human doctor did not depend on their feelings of power (*M*_high_ = 7.45, SD = 1.04; *M*_control_ = 7.53, SD = 0.99, *F* (1, 596) = 0.22, *p* = 0.64, ηp^2^ < 0.001; see Figure 3). Since more favorable attitudes toward the AI doctor indicate reduced resistance to AI care, this pattern suggests that power attenuated AI aversion.

#### 3.2.3 Discussion

Study 2 replicated the findings of the previous study, showing that a heightened sense of power increases acceptance of AI service providers but does not influence perceptions of human doctors. By comparing a high-power condition with a control group, this study isolated the unique effects of high power from low power. Additionally, the use of a semantic manipulation of power enhanced the marketing relevance of the findings. Building on these results, the next study further investigates the mediating role of decreased emotional thinking reliance through a moderated mediation approach.

### 3.3 Study 3: mechanisms of power on AI aversion

Study 3 tested the proposed process using a moderation-of-process approach (Spencer et al., 2005), leveraging theoretically relevant contexts (financial vs. dating decisions) that systematically vary the salience of emotional versus rational considerations. This design allows us to infer the proposed mechanism indirectly by examining whether the effect of power on AI acceptance is attenuated when affective input is naturally low. Building on our theorizing, we argue that the effect of power on AI aversion depends on the decision context. Specifically, a sense of power reduces AI aversion by decreasing individuals’ reliance on emotional thinking. In service domains that inherently emphasize rational decision-making—such as financial services—individuals are already primed to engage in more rational evaluations, thereby diminishing the incremental effect of power. In contrast, in service domains typically viewed as emotional—such as dating services—AI aversion is more pronounced. In these contexts, experiencing higher power is expected to attenuate AI aversion by shifting individuals toward more rational, outcome-focused evaluations. We predicted that in financial service contexts, the manipulation of power would not significantly alter individuals’ attitudes toward AI, whereas in dating service contexts, high-power participants would exhibit lower AI aversion compared to low-power participants.

#### 3.3.1 Procedures

We recruited 400 participants through Credamo with monetary compensation, and we excluded 35 participants who failed the attention check. Thus, the final sample size for study 3 was 365 (*M*_*a*ge_ = 28.45 years, SD = 7.54; 34% men, 66% women). They were randomly assigned to groups using a 2 (Power: high vs. low) × 2 (service type: financial vs. dating) between-subjects design. The service condition was pretested (*n* = 200), in which dating service was rated as highly emotional (*M*_date_ = 5.25, SD = 1.34 versus *M*_financial_ = 2.58, SD = 1.14, *p* < 0.001) and financial service was rated as highly rational (*M*_date_ = 3.27, SD = 1.40 versus *M*_financial_ = 5.67, SD = 1.12, *p* < 0.001).

##### 3.3.1.1 Experimental conditions

Similar to study 1, we asked participants to complete a role-playing writing task. In the high-power condition, participants were asked to imagine themselves as the owner of a company and try to vividly imagine what they would be like as a boss (i.e., how they would feel, think, and act). By contrast, in the low-power condition, participants were asked to imagine themselves as the employee of a company and then finish the writing task. After finishing the writing task, participants were required to report their feelings during the imagination task as a manipulation check. We used three items on a 9-point Likert-type scale anchored at *1 = very powerless/obedient/anxious* and *9 = very powerful/dominant/confident*.

Regarding the service type, in the dating service, participants were told they were planning to find a date and required a dating consultant to recommend suitable candidates based on their needs. In financial services, they were told they were planning to invest a sum of money and required a financial advisor to provide a comprehensive financial plan.

##### 3.3.1.2 AI acceptance measurements

After the service situation description, we asked participants to report their acceptance of the AI service provider on a 9-point Likert-scale, in which *1 = Totally unaccepted* and *9 = Totally accepted*. Demographic information was collected at the end as the control.

#### 3.3.2 Results

##### 3.3.2.1 Manipulation check

The manipulation check of power consisted of three items, and the value for Cronbach’s *α* = 0.956. A one-way analysis of variance (ANOVA) of the power manipulation on participants’ current feeling of power was significant (*F* (1, 363) = 1778.04, *p* < 0.001). Specifically, people who imagined themselves as a boss felt more powerful (*M*_powerful_ = 7.83, SD = 0.85) than people who imagined themselves as an employee (*M*_powerless_ = 2.76, SD = 1.37). Thus, our manipulation of the sense of power was successful.

##### 3.3.2.2 AI acceptance

**Figure 4:**
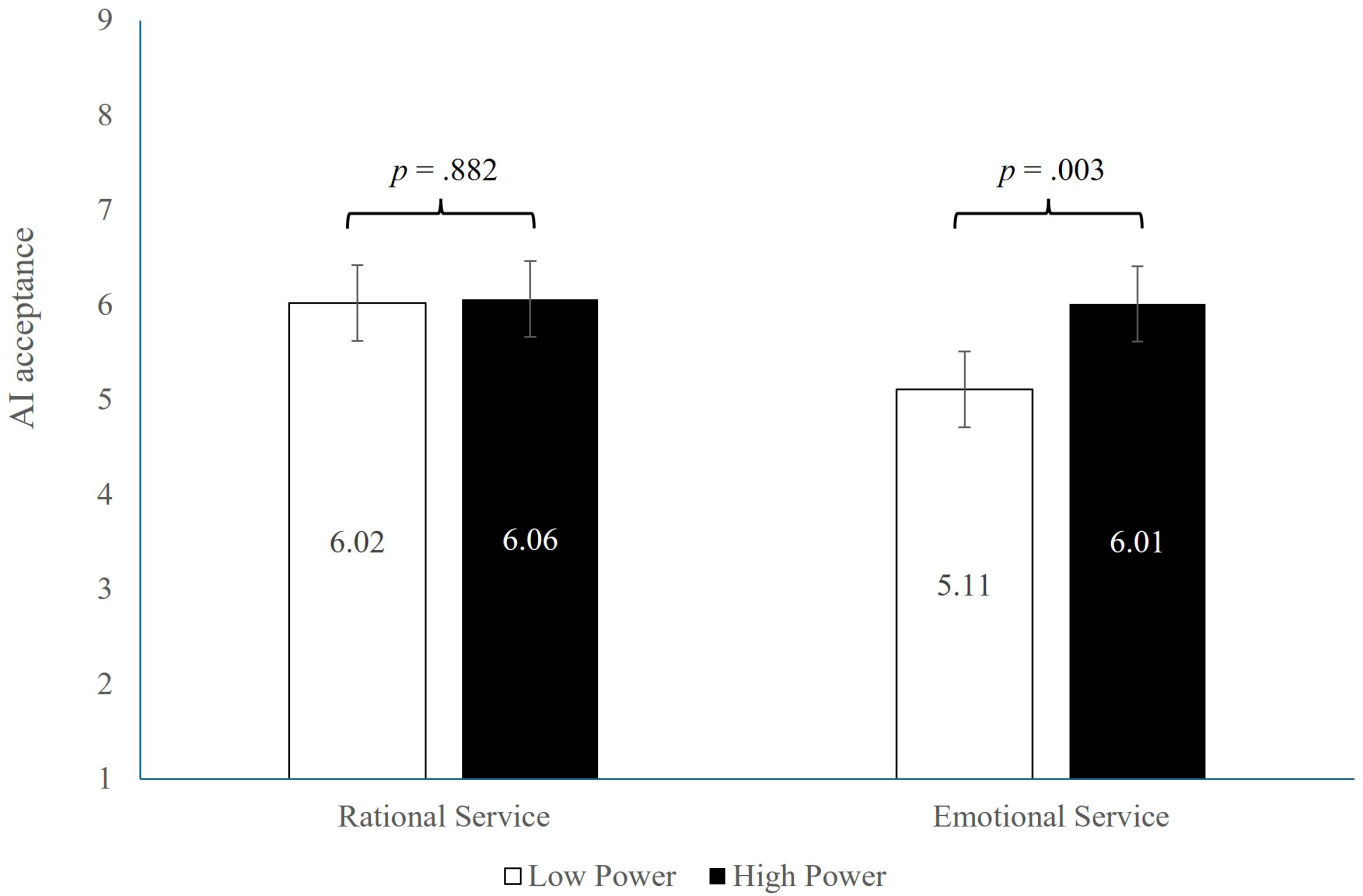
Study 3 result.

Next, we tested the moderating effect of service type using Model 1 in the PROCESS (Hayes, 2017), with sense of power as the independent variable (0 = lower power, 1 = high power), service type as the moderator (0 = emotional, 1 = rational), and acceptance of AI server as the dependent variable. After controlling the main effect of feeling of power (*β* = 0.89, *t* = 2.98, *p* = 0.003), the effect of service type (*β* = 0.91, t = 3.05, *p* = 0.002), we found a significant interaction between feeling of power and service type on acceptance of AI server (*β* = −0.85, *t* = −2.02, *p* = 0.043, *R*^2^ = 0.011). As predicted, in the emotional service condition, participants who felt powerful reported greater acceptance of AI than those who felt powerless (*M*_high_ = 6.01, SD = 2.10 vs. *M*_low_ = 5.11, SD = 2.16, *p* = 0.003), where higher values indicate greater AI acceptance. In contrast, in the rational service condition, sense of power did not affect AI acceptance (*M*_high_ = 6.06, SD = 1.93 vs. *M*_low_ = 6.02, SD = 1.84, *p* = 0.882; see Figure 4).

#### 3.3.3 Discussion

Study 3 employed a moderation-of-process approach to test the proposed mechanism, thereby offering a deeper insight into how a sense of power shapes participants’ acceptance of AI services. To do so, we strategically contrasted decision contexts that naturally evoke rational (financial decision) versus emotional (dating decision) processing and provided a rigorous validation of the psychological process. In the financial context, sense of power did not influence AI server evaluation, and both high- and low-power participants showed similarly high acceptance of AI. In contrast, in the emotionally rich dating context, high-power individuals exhibited significantly greater acceptance of AI than low-power individuals. This divergence across contexts provides convergent evidence for our proposed mechanism: feeling powerful reduces reliance on emotional decision-making, which in turn attenuates AI aversion. Thus, H_2_ and H_3_ are supported. Overall, the findings reinforce the central argument that power shapes AI acceptance by shifting individuals’ reliance on emotion during judgment.

## 4 Theoretical and managerial contributions

### 4.1 Research conclusion

Across three studies, we demonstrate that a heightened sense of power reduces individuals’ aversion to AI services. This effect emerges when power is elicited both explicitly and implicitly and is observed across diverse service contexts, such as medical care, finance, and dating, with its magnitude depending on the emotional versus rational nature of the decision context. Importantly, the effect is robust across multiple operationalizations of AI aversion, such as relative preference for human versus AI providers, separate evaluations of AI and human providers, and direct measures of AI acceptance. We propose that power attenuates AI aversion by reducing reliance on emotional decision-making. Consistent with this account, we find that power has little impact in service domains that already minimize affective input, but it significantly mitigates AI aversion in emotionally laden contexts. Beyond advancing theoretical understanding of the psychological processes underlying AI adoption, our findings highlight that subtle power-enhancing cues (e.g., addressing individuals with honorifics) can increase perceived power and thereby promote openness to AI services, offering actionable implications for service design.

### 4.2 Theoretical contributions

Our research contributes to the literature on AI adoption and consumer behavior by introducing a novel situational determinant of AI aversion: individuals’ sense of power. Prior efforts to reduce AI aversion have largely focused on enhancing AI’s functional attributes—such as improving intelligence (Hou et al., 2024), transparency (Cadario et al., 2021; Mourali et al., 2025), or personalization (Chen et al., 2024; Longoni et al., 2019)—and increasing user control (Dietvorst et al., 2018; Shanks et al., 2024). While these approaches are valuable, they often overlook the role of individuals’ psychological states, which directly shape how people evaluate and interact with AI. We demonstrate that a heightened sense of power—situationally elicited and independent of AI’s inherent attributes—reduces AI aversion by shifting how individuals weigh affective versus instrumental considerations during evaluation.

Beyond AI research, our findings also enrich the literature on power. Whereas prior study has often emphasized the negative interpersonal consequences of power, such as increased selfishness or reduced perspective taking (Galinsky et al., 2006), we show that power can generate constructive downstream consequences in AI service contexts. Specifically, when considering AI services, individuals who feel powerful place less weight on the provider’s identity as human versus non-human and focus more on instrumental outcomes, thereby attenuating species-based differences in evaluation. This perspective broadens the theoretical understanding of power, highlighting boundary conditions under which power fosters constructive outcomes, and offering marketers actionable insights for promoting AI adoption.

Third, our findings are particularly important because many service contexts where AI is increasingly adopted are inherently emotion-driven (e.g., health care, counseling, or personal relationships). Prior research suggests that individuals perceive subjective, emotion-laden tasks as more suitable for humans, whereas objective, analytic tasks are viewed as more appropriate for machines (e.g., Castelo et al., 2019). Our results extend this literature by demonstrating that situational psychological states, such as power, can shift these perceptions, enabling individuals to evaluate AI services more favorably even in domains traditionally dominated by emotional considerations.

### 4.3 Managerial implications

Our findings offer several practical implications for marketers, service designers, and policymakers. First, service providers can leverage subtle contextual cues to elevate individuals’ sense of power and thereby reduce resistance to AI services. For example, language styles that employ honorifics or respectful forms of address, as demonstrated in our studies, can enhance individuals’ perceived power. Similarly, service interfaces and AI agents may incorporate design elements that signal respect or deference, which could foster users’ sense of empowerment.

Second, from a policy perspective, regulators and public institutions could use these insights to encourage AI adoption in socially beneficial domains (e.g., healthcare and education). Campaigns emphasizing consumer agency and empowerment may prove more effective than those solely highlighting AI’s technical capabilities.

Finally, firms should recognize that the effectiveness of AI adoption strategies depends not only on improving AI systems themselves but also on shaping the psychological context in which individuals engage with them. Attending to individuals’ sense of power may serve as a low-cost, scalable intervention for increasing openness to AI-based services.

## 5 Research limitations and future research directions

Several limitations open avenues for future research. First, the form of AI may influence our effects. While we conceptualize AI as a categorical “other” that elicits emotional discomfort—and show that power attenuates this discomfort—the magnitude of this process may depend on how AI is instantiated. Embodied (vs. invisible) AI may heighten discomfort by making AI’s otherness more salient, and anthropomorphism may further modulate whether AI is construed as a social actor versus a tool. Future research could test these form-based boundary conditions.

Second, although the present studies were conducted in a Chinese context, the psychological mechanisms identified here may offer broader insight into responses to AI service providers in other regions where AI services are rapidly expanding. Nevertheless, the generalizability of our findings beyond China should be interpreted with caution. Cultural norms surrounding hierarchy, authority, and interpersonal relations may shape how power influences AI aversion. For instance, in higher power-distance societies, where power is more strongly associated with status and social control, its effect on AI aversion may be more pronounced. This consideration also underscores the global relevance of examining when and where the present effects may or may not generalize, particularly as AI services expand across diverse cultural and institutional settings. More broadly, cross-national differences in regulatory regimes, privacy norms, media discourse, and the availability and quality of AI-enabled services may influence baseline trust in AI, perceived risk, and the perceived viability of AI as an alternative to human providers. Accordingly, the psychological processes documented here may not manifest uniformly across contexts. Future research could test the robustness of these effects across countries and regions using matched service settings and comparable AI implementations.

Third, future research should examine the potential downsides of increased AI adoption. Reduced aversion is not unambiguously beneficial: broader acceptance of AI may also introduce ethical and societal risks that warrant careful attention. One concern is algorithmic bias (Bracic et al., 2022), arising from biased data, imperfect measurement, or model limitations, which may silently exacerbate inequities, especially when greater comfort with AI dampens scrutiny and makes such problems harder to detect. Another concern is AI dependence (Vasconcelos et al., 2023): as people become more willing to rely on AI, they may defer to algorithmic recommendations, reduce independent judgment, and show less critical scrutiny. Beyond these issues, scholars have also raised broader concerns about privacy risks, the potential erosion of human relationships, and the gradual degradation of human skills as individuals increasingly outsource cognitive or relational tasks to intelligent systems (Valčo, 2024; Vuong and Ho, 2025). Future studies could examine when these risks emerge and identify safeguards that foster calibrated trust in AI (e.g., accountability cues, bias audits, privacy protection mechanisms, and human-in-the-loop review).

Finally, an additional avenue for future research is to test this theoretical connection more directly. Although our framework suggests that AI may be experienced as a categorical “other,” we did not directly measure this process in the present studies. This was partly intentional: the proposed reaction is likely to be relatively automatic, and direct self-reports may not fully capture it if participants lack introspective access or engage in *post hoc* correction. Instead, we adopted an indirect strategy that focused on the behavioral implications of the proposed process and tested it through theoretically relevant boundary conditions. An advantage of this approach is that it allows the mechanism to be evaluated without relying exclusively on participants’ explicit reports of their own affective reactions. Future research could complement this strategy with more direct measures by examining whether responses to AI share psychological features with intergroup processes or whether activating intergroup thinking increases AI aversion. Such study would help clarify the extent to which aversion to AI reflects broader social–cognitive responses to socially distinct non-human agents.

## Data Availability

The original contributions presented in the study are included in the article/Supplementary material, further inquiries can be directed to the corresponding author.

## Footnotes

1 Aidox, https://www.aidoc.com/.

2 Babylon Health, https://digitalhealth.london/innovation-directory/profile/babylon-health.

3 Betterment, https://www.betterment.com/.

4 Wealthfront, https://www.wealthfront.com/.

5 Tinder, https://tinder.com/.

6 Hinge, https://hinge.co/en-gb.

7
https://www.credamo.com/
