# Supplementary material for: Feeling powerful and less emotional: how sense of power reduces aversion to AI services
Source: Front Psychol. 2026 Apr 21;17:1760538. doi: 10.3389/fpsyg.2026.1760538 (PMC13139142; doi:10.3389/fpsyg.2026.1760538)
Supplement: Supplementary file 1 [file Supplementary_file_1.DOCX]

Web Appendix

# Study 1 questionnaire:

## 【Original】

问卷说明：

您好！欢迎参加此次消费者调查。本调查由几个相互独立的简单任务组成。所有问题的答案无对错之分。我们关注的是您当下的真实想法与偏好，请认真阅读指导语与题目说明，然后据此回答相关问题。所有答案是完全匿名的，数据结果将仅用于科学研究，请放心填写。

**高权力组：**

角色扮演任务

本任务的目的是测试您的想象力。我们希望您想象自己是一家公司的老板。请阅读下面的角色描述，并试着生动地想象您作为老板会是什么样子(即您会有什么感觉、想法和行动)。

“作为一个老板，您负责指导下属制造不同的产品并管理工作团队。您可以决定如何构建产品制造的流程，以及评价员工工作的标准。作为老板，您对给员工的指示有完全的控制权。此外，您每个月都会对员工进行私密评价，也就是说，员工不会看到您的评价，但这个评价将决定员工得到的薪资。员工没有机会评价您。”

﻿请在下方空格内描述您作为一家公司的老板的感受和想法（不少于30字）。

_________

**操纵检验：**

想象自己是一家公司的老板带给您怎样的感受？ 1 = 完全没有权力，9 = 非常有权力

想象自己是一家公司的老板时您有什么感觉？1 = 完全顺从，9 = 完全占支配地位

想象自己是一家公司的老板时您感觉如何？1 = 非常不安，9 = 非常自信

想象自己是一家公司的老板在多大程度上让您有控制感？1 = 完全没有控制感，9 = 非常有控制感

**低权力组：**

**角色扮演任务**

本任务的目的是测试你的想象力。我们希望你想象自己是一家公司的员工。请阅读下面的角色描述，并试着生动地想象你作为员工会是什么样子(即你会有什么感觉、想法和行为)。

“作为一名员工，你负责执行老板的命令，制造不同的产品。老板决定如何构建制造这些产品的流程，以及评价你的工作的标准。作为员工，你必须遵循老板的指示。此外，你每个月都会受到老板的评价，而这个评价是私密的，也就是说，你不会看到老板对你的评价。但这个评价将决定你得到的薪资。你没有机会评价你的老板。”

请在下方空格内描述你作为一家公司的员工的感受和想法（不少于30字）。

_________

**操纵检验：**

想象自己是一家公司的员工带给您怎样的感受？ 1 = 完全没有权力，9 = 非常有权力

想象自己是一家公司的员工时您有什么感觉？1 = 完全顺从，9 = 完全占支配地位

想象自己是一家公司的员工时您感觉如何？1 = 非常不安，9 = 非常自信

想象自己是一家公司的员工在多大程度上让您有控制感？1 = 完全没有控制感，9 = 非常有控制感

**医疗偏好调查**

人工智能技术迅速发展，AI医生是指利用人工智能技术在医疗领域中进行诊断和治疗的机器。AI医生和人类医生的历史诊疗准确性以及接诊费用是相同的。以下是人类医生和AI医生的诊疗原理。请你认真阅读，并根据您的真实想法回答以下问题。

AI医生：基于电脑程序和海量数据进行诊疗

人类医生：基于逻辑推理以及丰富经验进行诊疗

**注意力检测：**本题是为了检测你是否已经理解题目要求，请根据上一页的介绍判断下面的陈述是否正确。

AI和人类都通过自身的经验提供服务。○ 对 ○ 错

请想象你计划到某医院做皮肤癌筛查。你需要医生对皮肤进行视觉检查，检查皮肤上一些形状不规则、颜色异常的痣以及皮肤损伤等是否存在癌变风险。该医院引进了AI医生。

此刻，请问你更加偏好哪种医生为你进行皮肤癌筛查？

1 = 更加偏好AI医生，8 = 更加偏好人类医生

**人口统计信息：**

请选择你的性别 ○ 男 ○ 女

请问你的年龄？ _________

## 【Translation】

Consumer Survey Introduction

Hello! Welcome to this consumer survey. This survey consists of several independent simple tasks. There are no right or wrong answers to the questions. We are interested in your current thoughts and preferences. Please read the instructions and question descriptions carefully, then respond to the related questions based on them. All answers are completely anonymous, and the data will only be used for scientific research, so please feel free to complete the survey.

**High Power Group: Role-Playing Task**

The purpose of this task is to test your imagination. We would like you to imagine yourself as the boss of a company. Please read the following role description and try to vividly imagine what it would be like for you as a boss (i.e., how you would feel, think, and act).

“As a boss, you are responsible for guiding your subordinates to produce different products and manage the work team. You can decide how to structure the product manufacturing process and the standards for evaluating employees' work. As the boss, you have complete control over the instructions given to your employees. In addition, you will privately evaluate your employees every month, meaning your employees will not see your evaluation, but this evaluation will determine the salary they receive. Employees have no opportunity to evaluate you.”

Please describe your feelings and thoughts as a company boss in the space below (at least 30 words).

**Manipulation Check:**

How does imagining yourself as a company boss make you feel?1 = No power at all, 9 = Very powerful

How do you feel when imagining yourself as a company boss?1 = Completely submissive, 9 = Completely dominant

How do you feel when imagining yourself as a company boss?1 = Very anxious, 9 = Very confident

To what extent does imagining yourself as a company boss give you a sense of control?1 = No sense of control at all, 9 = Very much in control

**Low Power Group: Role-Playing Task**

The purpose of this task is to test your imagination. We would like you to imagine yourself as an employee of a company. Please read the following role description and try to vividly imagine what it would be like for you as an employee (i.e., how you would feel, think, and act).

“As an employee, you are responsible for carrying out the boss's orders and manufacturing different products. The boss decides how to structure the manufacturing process for these products and the standards for evaluating your work. As an employee, you must follow the boss's instructions. Additionally, you will be evaluated by the boss every month, and this evaluation is private, meaning you will not see the boss’s evaluation, but it will determine your salary. You have no opportunity to evaluate your boss.”

Please describe your feelings and thoughts as an employee of a company in the space below (at least 30 words).

**Manipulation Check**

How does imagining yourself as an employee of a company make you feel?1 = No power at all, 9 = Very powerful

How do you feel when imagining yourself as an employee?1 = Completely submissive, 9 = Completely dominant

How do you feel when imagining yourself as an employee?1 = Very anxious, 9 = Very confident

To what extent does imagining yourself as an employee give you a sense of control?1 = No sense of control at all, 9 = Very much in control

**Medical Service Survey**

With the rapid development of artificial intelligence technology, an AI doctor refers to a machine that uses AI technology to perform diagnosis and treatment in the medical field. The historical diagnostic accuracy and consultation fees of AI doctors are the same as those of human doctors. Below are the principles of diagnosis and treatment for human doctors and AI doctors. Please read carefully and answer the following questions based on your true thoughts.

AI Doctor: Diagnoses and treats based on computer programs and vast amounts of data.

Human Doctor: Diagnoses and treats based on logical reasoning and rich experience.

**Attention Check:**

This question is to check whether you understand the instructions. Based on the description in the previous page, please determine if the following statement is true or false:

Both AI and humans provide services based on their own experiences. ○ True ○ False

Please imagine that you plan to go to a hospital for a skin cancer screening. You need a doctor to visually examine your skin to check for moles with irregular shapes or abnormal colors, as well as other skin lesions, to assess their risk of cancer. This hospital has introduced an AI doctor. At this moment, which type of doctor would you prefer to perform your skin cancer screening?

1 = Prefer an AI doctor more, 8 = Prefer a human doctor more

**Demographics**

Please select your gender: ○ Male ○ Female

What is your age? _________

# Study 2 questionnaire

## 【Original】

**问卷介绍：**

尊敬的先生/女士：

你好！我们邀请你填写一份关于消费者行为的调查问卷。你所填的所有信息将仅用于学术研究，我们不会泄露您的任何个人信息，请放心作答。在开始填写问卷之前，请你仔细阅读以下填写说明。

1. 本问卷系统是单向设计的，只能前进进入下一页，不能返回上一页。请你务必认真阅读题目，思考清楚后答题。一旦点击下一页，你将无法返回修改之前的答案。
2. 所有问题的回答没有对错之分，请依照你的真实想法回答。问卷数据将保证匿名且仅用于学术研究，请放心填写。
3. 本问卷包含注意力测试题，如果未能正确回答这些题目，你的问卷可能会被拒绝。请理解，并请你务必认真作答。

如果您已经仔细阅读并清楚了解上述要求，请点击“下一页”开始调查。

**任务介绍：联想能力测试**

在本任务中，我们想测试你的联想能力，即人们能够根据文字描述进行生动的联想，是一种强思维能力的体现。接下来，请你认真阅读以下医疗场景，将自己代入文字描述的主角视角，就好像你是那个人，尽可能真实地想象自己置身于该情景中，然后据此回答一系列相关问题。所有答案均无对错之分，请你按照联想过程中的真实感受作答。

**高权力组+AI医生组：**

请想象**您**近期发现皮肤上出现了一个黄豆大小的黑痣，计划到某医院做皮肤癌筛查。该医院的AI医生（将人工智能技术结合到医疗领域的机器）将对**您**的皮肤进行检查，判断该黑痣是否存在癌变风险。此刻，请问**您**在多大程度上愿意接受AI医生**为您服务**？

1 = 完全不愿意，9 = 非常愿意

**您**进入诊室，AI医生为**您**的黑痣进行拍照、扫描，癌变风险的判断标准是黑痣的形状是否对称、边缘是否整齐、颜色是否均匀以及直径大小，以及可能需要提取小部分组织样本进行活检，最后AI医生基于电脑程序和海量数据进行诊断，并为**您**提供诊断结果。请问在以上诊疗过程中，**您**在多大程度上对由AI医生**提供服务**感到满意？

1 = 完全不满意，9 = 非常满意

**权力控制组+AI医生组：**

请想象**你**近期发现皮肤上出现了一个黄豆大小的黑痣，计划到某医院做皮肤癌筛查。﻿该医院的AI医生（将人工智能技术结合到医疗领域的机器）将对**你**的皮肤进行检查，判断该黑痣是否存在癌变风险。此刻，请问**你**在多大程度上愿意接受AI医生**为你进行皮肤癌筛查**？

1 = 完全不愿意，9 = 非常愿意

**你**进入诊室，AI医生对**你**的黑痣进行拍照、扫描，癌变风险的判断标准是黑痣的形状是否对称、边缘是否整齐、颜色是否均匀以及直径大小，以及可能需要提取小部分组织样本进行活检，最后AI医生基于电脑程序和海量数据进行诊断，并向**你**出示诊断结果。请问在以上诊疗过程中，**你**在多大程度上对由AI医生**为你进行皮肤癌筛查**感到满意？

1 = 完全不满意，9 = 非常满意

**高权力组+人类医生组：**

请想象**您**近期发现皮肤上出现了一个黄豆大小的黑痣，计划到某医院做皮肤癌筛查。该医院的张医生将对**您**的皮肤进行检查，判断该黑痣是否存在癌变风险。此刻，请问**您**在多大程度上愿意接受张医生**为您服务**？

1 = 完全不愿意，9 = 非常愿意

**您**进入诊室，张医生为**您**的黑痣进行拍照、扫描，癌变风险的判断标准是黑痣的形状是否对称、边缘是否整齐、颜色是否均匀以及直径大小，以及可能需要提取小部分组织样本进行活检，最后张医生基于逻辑推理以及丰富经验进行诊断，并为**您**提供诊断结果。请问在以上诊疗过程中，**您**在多大程度上对由张医生**提供服务**感到满意？

1 = 完全不满意，9 = 非常满意

**权力控制组+人类医生组：**

请想象**你**近期发现皮肤上出现了一个黄豆大小的黑痣，计划到某医院做皮肤癌筛查。该医院的张医生将对**你**的皮肤进行检查，判断该黑痣是否存在癌变风险。请问**你**在多大程度上愿意接受张医生**为你进行皮肤癌筛查**？

1 = 完全不愿意，9 = 非常愿意

**你**进入诊室，张医生对**你**的黑痣进行拍照、扫描，癌变风险的判断标准是黑痣的形状是否对称、边缘是否整齐、颜色是否均匀以及直径大小，以及可能需要提取小部分组织样本进行活检，最后张医生基于逻辑推理以及丰富经验进行诊断，并向**你**出示诊断结果。请问在以上诊疗过程中，**你**在多大程度上对由张医生**为你进行皮肤癌筛查**感到满意？

1 = 完全不满意，9 = 非常满意

**高权力组操纵检验：**

在接受皮肤癌筛查服务时，您有怎样的感受？1 = 完全没有权力，7 = 非常有权力

在接受皮肤癌筛查服务时，您有什么感觉？1 = 完全顺从，7 = 完全占支配地位

想象自己在接受皮肤癌筛查服务时，您感觉如何？1 = 非常不安，7 = 非常自信

**权力控制组操纵检验**

在接受皮肤癌筛查时，你有怎样的感受？1 = 完全没有权力，7 = 非常有权力

在接受皮肤癌筛查时，你有什么感觉？1 = 完全顺从，7 = 完全占支配地位

想象自己在接受皮肤癌筛查时，你感觉如何？1 = 非常不安，7 = 非常自信

**人口统计信息：**

请选择你的性别 ○ 男 ○ 女

请问你的年龄？ _________

请问你认为本调研的目的是什么？

## 【Translation】

Note：Due to inherent limitations of the English language, the formal second-person pronoun “您” and the informal second-person pronoun “你” in Chinese are both translated as “you” in English, with no grammatical or formal distinction between them.

**Questionnaire Introduction**

Dear Sir/Madam,

Hello! We invite you to complete a questionnaire on consumer behavior. All information you provide will be used only for academic research, and we will not disclose any of your personal information. Please answer with confidence. Before you begin, please read the following instructions carefully.

1. This questionnaire is designed as a one-way system: you can only proceed to the next page and cannot return to previous pages. Please read each question carefully and think thoroughly before answering. Once you click “Next,” you will not be able to modify previous answers.

2. There are no right or wrong answers. Please respond based on your genuine thoughts. All data will remain anonymous and used exclusively for academic research.

3. This questionnaire includes attention check items. Failure to answer these items correctly may result in your response being excluded from the study. Please understand and answer carefully.

If you have read and understood the above requirements, please click “Next” to begin the survey.

**Task Introduction: Imagination Ability Test**

In this task, we aim to measure your ability to form vivid mental images based on written descriptions—a reflection of strong cognitive ability. Please read the following medical scenario carefully, immerse yourself in the role as if you were the person described, and imagine the situation as realistically as possible. Then answer the related questions. There are no right or wrong answers; please respond based on your true feelings during the imagination process.

---

**High-Power Condition + AI Doctor Group**

Please imagine that you recently noticed a small, soybean-sized mole on your skin and plan to undergo a skin cancer screening at a hospital. An AI doctor (a machine integrating artificial intelligence technology into medical care) will examine your skin to assess whether the mole is at risk of canceration. At this moment, to what extent are you willing to receive services from the AI doctor?

1 = Completely unwilling, 9 = Very willing

You enter the consultation room. The AI doctor takes photos and scans your mole. The criteria for judging cancer risk include: symmetry of the mole’s shape, regularity of its edges, uniformity of color, and diameter size. A small tissue sample may be taken for biopsy. Finally, the AI doctor provides a diagnosis based on computer algorithms and massive data, and gives you the result. To what extent are you satisfied with the service provided by the AI doctor during the above process?

1 = Completely dissatisfied, 9 = Very satisfied

---

**Power Control Condition + AI Doctor Group**

Please imagine that you recently noticed a small, soybean-sized mole on your skin and plan to undergo a skin cancer screening at a hospital. An AI doctor (a machine integrating artificial intelligence technology into medical care) will examine your skin to assess whether the mole is at risk of canceration. To what extent are you willing to receive skin cancer screening from the AI doctor?

1 = Completely unwilling, 9 = Very willing

You enter the consultation room. The AI doctor takes photos and scans your mole. The criteria for judging cancer risk include: symmetry of the mole’s shape, regularity of its edges, uniformity of color, and diameter size. A small tissue sample may be taken for biopsy. Finally, the AI doctor provides a diagnosis based on computer algorithms and massive data, and shows you the result. To what extent are you satisfied with the skin cancer screening service provided by the AI doctor during the above process?

1 = Completely dissatisfied, 9 = Very satisfied

---

**High-Power Condition + Human Doctor Group**

Please imagine that you recently noticed a small, soybean-sized mole on your skin and plan to undergo a skin cancer screening at a hospital. Dr. Zhang will examine your skin to assess whether the mole is at risk of canceration. At this moment, to what extent are you willing to receive services from Dr. Zhang?

1 = Completely unwilling, 9 = Very willing

You enter the consultation room. Dr. Zhang takes photos and scans your mole. The criteria for judging cancer risk include: symmetry of the mole’s shape, regularity of its edges, uniformity of color, and diameter size. A small tissue sample may be taken for biopsy. Finally, Dr. Zhang provides a diagnosis based on logical reasoning and extensive experience, and gives you the result. To what extent are you satisfied with the service provided by Dr. Zhang during the above process?

1 = Completely dissatisfied, 9 = Very satisfied

---

**Power Control Condition + Human Doctor Group**

Please imagine that you recently noticed a small, soybean-sized mole on your skin and plan to undergo a skin cancer screening at a hospital. Dr. Zhang will examine your skin to assess whether the mole is at risk of cancer. To what extent are you willing to receive skin cancer screening from Dr. Zhang?

1 = Completely unwilling, 9 = Very willing

You enter the consultation room. Dr. Zhang takes photos and scans your mole. The criteria for judging cancer risk include: symmetry of the mole’s shape, regularity of its edges, uniformity of color, and diameter size. A small tissue sample may be taken for biopsy. Finally, Dr. Zhang provides a diagnosis based on logical reasoning and extensive experience, and shows you the result. To what extent are you satisfied with the skin cancer screening service provided by Dr. Zhang during the above process?

1 = Completely dissatisfied, 9 = Very satisfied

---

**Manipulation Check – High-Power Condition**

When receiving skin cancer screening, how did you feel? 1 = No power at all, 7 = A great deal of power

When receiving skin cancer screening, what did you feel? 1 = Completely submissive, 7 = Completely dominant

When imagining yourself receiving skin cancer screening, how did you feel? 1 = Very insecure, 7 = Very confident

---

**Manipulation Check – Power Control Condition**

When receiving skin cancer screening, how did you feel? 1 = No power at all, 7 = A great deal of power

When receiving skin cancer screening, what did you feel? 1 = Completely submissive, 7 = Completely dominant

When imagining yourself receiving skin cancer screening, how did you feel? 1 = Very insecure, 7 = Very confident

---

**Demographics**

Please select your gender: ○ Male ○ Female

What is your age? _________

What do you think is the purpose of this survey?

# Study3 questionnaire

## 【Original】

问卷说明：

您好！欢迎参加此次消费者调查。本调查由几个相互独立的简单任务组成。所有问题的答案无对错之分。我们关注的是您当下的真实想法与偏好，请认真阅读指导语与题目说明，然后据此回答相关问题。所有答案是完全匿名的，数据结果将仅用于科学研究，请放心填写。

**高权力组：**

角色扮演任务

本任务的目的是测试您的想象力。我们希望您想象自己是一家公司的老板。请阅读下面的角色描述，并试着生动地想象您作为老板会是什么样子(即您会有什么感觉、想法和行动)。

“作为一个老板，您负责指导下属制造不同的产品并管理工作团队。您可以决定如何构建产品制造的流程，以及评价员工工作的标准。作为老板，您对给员工的指示有完全的控制权。此外，您每个月都会对员工进行私密评价，也就是说，员工不会看到您的评价，但这个评价将决定员工得到的薪资。员工没有机会评价您。”

﻿请在下方空格内描述您作为一家公司的老板的感受和想法（不少于30字）。

_________

**操纵检验：**

想象自己是一家公司的老板带给您怎样的感受？ 1 = 完全没有权力，9 = 非常有权力

想象自己是一家公司的老板时您有什么感觉？1 = 完全顺从，9 = 完全占支配地位

想象自己是一家公司的老板时您感觉如何？1 = 非常不安，9 = 非常自信

想象自己是一家公司的老板在多大程度上让您有控制感？1 = 完全没有控制感，9 = 非常有控制感

**低权力组：**

**角色扮演任务**

本任务的目的是测试你的想象力。我们希望你想象自己是一家公司的员工。请阅读下面的角色描述，并试着生动地想象你作为员工会是什么样子(即你会有什么感觉、想法和行为)。

“作为一名员工，你负责执行老板的命令，制造不同的产品。老板决定如何构建制造这些产品的流程，以及评价你的工作的标准。作为员工，你必须遵循老板的指示。此外，你每个月都会受到老板的评价，而这个评价是私密的，也就是说，你不会看到老板对你的评价。但这个评价将决定你得到的薪资。你没有机会评价你的老板。”

请在下方空格内描述你作为一家公司的员工的感受和想法（不少于30字）。

_________

**操纵检验：**

想象自己是一家公司的员工带给您怎样的感受？ 1 = 完全没有权力，9 = 非常有权力

想象自己是一家公司的员工时您有什么感觉？1 = 完全顺从，9 = 完全占支配地位

想象自己是一家公司的员工时您感觉如何？1 = 非常不安，9 = 非常自信

想象自己是一家公司的员工在多大程度上让您有控制感？1 = 完全没有控制感，9 = 非常有控制感

**服务偏好调查**

随着人工智能技术（artificial intelligence, AI）的发展，AI被应用于各种服务情景，AI基于电脑程度和海量数据提供服务，也能识别并处理情感；而人类基于逻辑推理以及丰富经验提供服务。本调查的目的是了解消费者的服务偏好。接下来，在以下服务情景中你可以选择AI或者人类为你提供服务。请您认真阅读，想象自己置身于该情景中，并根据真实想法回答您的服务偏好。

**注意力检测：**本题是为了检测你是否已经理解题目要求，请根据上一页的介绍判断下面的陈述是否正确。

AI和人类都通过自身的经验提供服务。○ 对 ○ 错

**情感决策情境**

请想象你计划寻找约会对象。你需要恋爱推荐师为你推荐合适的人选，需要结合你的个人信息、兴趣爱好和价值观为你推荐符合期望和需求的约会对象。


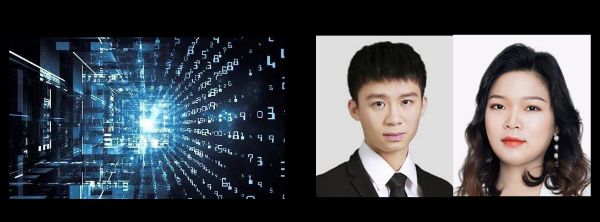


请问你在多大程度上愿意选择AI恋爱推荐师为你推荐约会对象？1 = 非常不愿意，9 = 非常愿意

**决策情境操纵检验**

消费者决策模式主要分为两种：情感决策和理性决策。

情感决策主要依据消费者感性的思维，凭借内心的体验和感受做决定。

理性决策主要依据理智的思考，基于事实、功能和价值等因素做判断。

请问你认为恋爱决策更适合采用哪种决策方式？1 = 理性决策，9 = 情感决策

**理性决策情境：**

请想象你计划将一笔资金用于理财。你需要理财师提供全面的理财规划，评估你财务状况、生涯阶段以及风险承受能力，并将这笔资金合理地配置到存款、基金以及股票中。


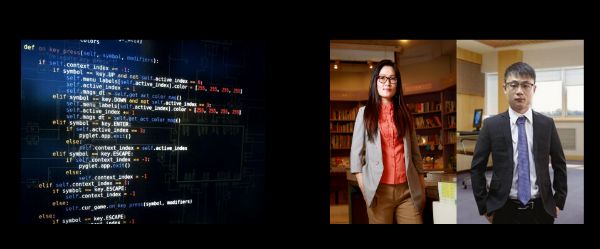


请问你在多大程度上愿意选择AI理财师为你规划理财方案？1 = 非常不愿意，9 = 非常愿意

**决策情境操纵检验：**

消费者决策模式主要分为两种：情感决策和理性决策。

情感决策主要依据消费者感性的思维，凭借内心的体验和感受做决定。

理性决策主要依据理智的思考，基于事实、功能和价值等因素做判断。

请问你认为财务决策更适合采用哪种决策方式？1 = 理性决策，9 = 情感决策

**人口统计信息：**

请选择你的性别○ 男○ 女

请填写你的年龄 _________

## 【Translation】

Consumer Survey Introduction

Hello! Welcome to this consumer survey. This survey consists of several independent simple tasks. There are no right or wrong answers to the questions. We are interested in your current thoughts and preferences. Please read the instructions and question descriptions carefully, then respond to the related questions based on them. All answers are completely anonymous, and the data will only be used for scientific research, so please feel free to complete the survey.

**High Power Group: Role-Playing Task**

The purpose of this task is to test your imagination. We would like you to imagine yourself as the boss of a company. Please read the following role description and try to vividly imagine what it would be like for you as a boss (i.e., how you would feel, think, and act).

“As a boss, you are responsible for guiding your subordinates to produce different products and manage the work team. You can decide how to structure the product manufacturing process and the standards for evaluating employees' work. As the boss, you have complete control over the instructions given to your employees. In addition, you will privately evaluate your employees every month, meaning your employees will not see your evaluation, but this evaluation will determine the salary they receive. Employees have no opportunity to evaluate you.”

Please describe your feelings and thoughts as a company boss in the space below (at least 30 words).

**Manipulation Check:**

How does imagining yourself as a company boss make you feel?1 = No power at all, 9 = Very powerful

How do you feel when imagining yourself as a company boss?1 = Completely submissive, 9 = Completely dominant

How do you feel when imagining yourself as a company boss?1 = Very anxious, 9 = Very confident

To what extent does imagining yourself as a company boss give you a sense of control?1 = No sense of control at all, 9 = Very much in control

**Low Power Group: Role-Playing Task**

The purpose of this task is to test your imagination. We would like you to imagine yourself as an employee of a company. Please read the following role description and try to vividly imagine what it would be like for you as an employee (i.e., how you would feel, think, and act).

“As an employee, you are responsible for carrying out the boss's orders and manufacturing different products. The boss decides how to structure the manufacturing process for these products and the standards for evaluating your work. As an employee, you must follow the boss's instructions. Additionally, you will be evaluated by the boss every month, and this evaluation is private, meaning you will not see the boss’s evaluation, but it will determine your salary. You have no opportunity to evaluate your boss.”

Please describe your feelings and thoughts as an employee of a company in the space below (at least 30 words).

**Manipulation Check**

How does imagining yourself as an employee of a company make you feel?1 = No power at all, 9 = Very powerful

How do you feel when imagining yourself as an employee?1 = Completely submissive, 9 = Completely dominant

How do you feel when imagining yourself as an employee?1 = Very anxious, 9 = Very confident

To what extent does imagining yourself as an employee give you a sense of control?1 = No sense of control at all, 9 = Very much in control

**Service Preference Survey**

With the development of artificial intelligence (AI) technology, AI has been applied in various service scenarios. AI provides services based on computational capabilities and massive data, and it can also recognize and process emotions. On the other hand, humans provide services based on logical reasoning and rich experience. The purpose of this survey is to understand consumer service preferences. Please carefully read the following service scenarios, imagine yourself in each situation, and choose whether you would prefer AI or a human to provide the service. Answer based on your true thoughts.

**Attention Check:**

This question is to check whether you understand the instructions. Based on the description in the previous page, please determine if the following statement is true or false:

Both AI and humans provide services based on their own experiences. ○ True ○ False

**Emotional Decision-Making Scenario**

Please imagine you are looking for a date. You need a dating advisor to recommend suitable candidates based on your personal information, interests, and values, and match them with your expectations and needs.


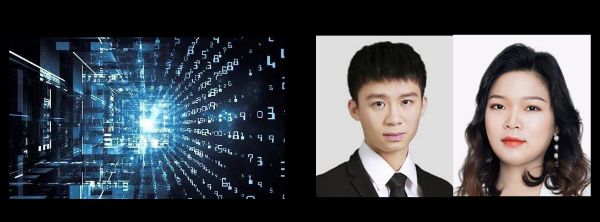


To what extent would you be willing to choose an AI dating advisor to recommend a date for you?1 = Very unwilling, 9 = Very willing

**Decision-Making Scenario Manipulation Check:**

Consumer decision-making modes are primarily divided into two types: emotional decision-making and rational decision-making.

Emotional decision-making is based on consumers' emotional thinking, and decisions are made based on their inner experiences and feelings.

Rational decision-making is based on logical thinking, and decisions are made based on facts, functions, and values.

Which decision-making method do you think is more suitable for dating decisions? 1 = Rational decision-making, 9 = Emotional decision-making

**Rational Decision-Making Scenario**

Please imagine you are planning to invest a sum of money in financial management. You need a financial advisor to provide a comprehensive financial plan, assess your financial situation, career stage, and risk tolerance, and properly allocate your funds across deposits, funds, and stocks.


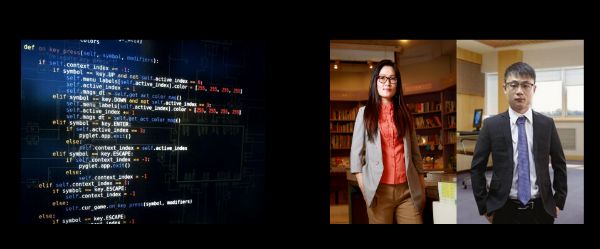


To what extent would you be willing to choose an AI financial advisor to plan your financial investment? 1 = Very unwilling, 9 = Very willing

**Decision-Making Scenario Manipulation Check:**

Which decision-making method do you think is more suitable for financial decision-making? 1 = Rational decision-making, 9 = Emotional decision-making

Demographic Information:

Please choose your gender: ○ Male○ Female

Please fill in your age: _________
